# Vestibular rehabilitation potential of commercially available virtual reality video games

**DOI:** 10.1186/s40463-023-00642-9

**Published:** 2023-08-21

**Authors:** Austin Heffernan, Lindsay Booth, Roland Fletcher, Desmond A. Nunez

**Affiliations:** 1 Division of Otolaryngology – Head and Neck Surgery, Department of Surgery, University of British Columbia, 2775 Laurel Street, 4Th Floor, Vancouver, BC V5Z 1M9 Canada; 2 Department of Physical Therapy, University of British Columbia, Vancouver, BC Canada; 3 Division of Otolaryngology – Head and Neck Surgery, Department of Surgery, Vancouver General Hospital, Vancouver, BC Canada

**Keywords:** Vestibular rehabilitation, Virtual reality, Head-mounted device, Video game, Vestibular hypofunction, Peripheral vestibular disorder, Physiotherapy

## Abstract

**Background:**

Peripheral vestibular disorders affect 2.8–6.5% of people. Standard treatment is vestibular rehabilitation therapy, and virtual reality (VR) could improve outcomes. The objective of this study was to identify the commercially available VR video game that is most congruent to vestibular rehabilitation therapy.

**Methods:**

A term search “virtual reality racing” was performed on the App Store in March 2022. Results were screened for free point-of-view racing games compatible with Android and iOS devices. An investigator was filmed playing each game and videos were distributed to 237 physiotherapists. Physiotherapists completed a survey of 5-point Likert scale questions that assessed the video games vestibular rehabilitation potential. Survey responses were analyzed using Friedman Two-Way ANOVA (alpha = 0.05) and paired samples sign test with Bonferroni correction.

**Results:**

The search yielded 58 games, 4 were included. Forty physiotherapists participated. VR Tunnel Race (VRTR) and VR Real World Bike Racing (VRWBR) had the greatest vestibular rehabilitation potential (median global scores = 18.00). VRTR replicated habituation exercises significantly (*p* < 0.001) better than Derby VR, and VRWBR replicated physiotherapist-prescribed exercises significantly (*p* < 0.001) better than VR X-Racer. There were no discernable significant differences between VRWBR and VRTR.

**Conclusions:**

VRTR and VRWBR are the most congruent VR games to standard vestibular rehabilitation. VRWBR is preferable to VRTR with respect to ease of use and the ability to alter the amount of optokinetic stimulation. Prospective studies are needed to confirm the efficacy of these videos games and to determine if they could be used as solitary treatments.

*Trial registration***:** Not applicable.

**Graphical abstract:**

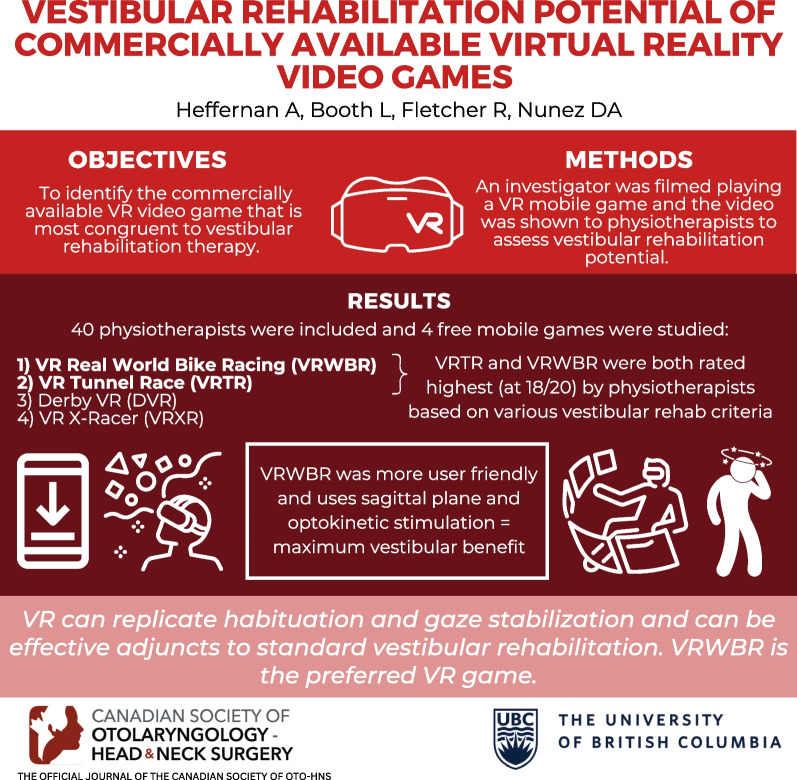

## Background

Peripheral vestibular disorders are highly morbid conditions that affect 2.8–6.5% of the population (women more frequently than men) and become more prevalent with age [1, 2]. The inner ear, specifically the vestibular apparatus and or its innervations are the site of pathology in these disorders. Dysfunction of different parts of the vestibular apparatus presents as several different peripheral vestibular disorders, including benign paroxysmal positional vertigo, Menière’s disease and vestibular neuritis. These can lead to symptoms of dizziness, imbalance, nausea, oscillopsia and occasional falling which can greatly reduce a patient’s quality of life [3]. In addition, the majority of patients with chronic symptoms develop depression and anxiety associated with their peripheral vestibular disorder [4, 5].

Current treatments for peripheral vestibular disorders are pharmacological, surgical, or physical including repositioning maneuvers and standard vestibular rehabilitation (SVR). The latter involves a range of exercises that include habituation, substitution, and adaptation exercises, where there is moderate evidence in support of virtual reality (VR) as a mode of delivering these exercises [6]. The interventions range from generic Cooksey Cawthorne to patient customized exercises [7]. These exert their effects by promoting adaptation of residual vestibular function, substitution of alternative strategies for lost vestibular function and habituation to unpleasant sensations [8, 9]. These mechanisms help to achieve the goals of SVR; namely to enhance postural stability, improve gaze, reduce vertigo, and improve the scope and scale of activities of daily living [10]. Despite these intentions, SVR is considered physical rehabilitation (PR), thus it is subject to the same factors that affect PR adherence including preconceptions of PR, perceived exertion during sessions, financial barriers, and inconvenience for the patient [11, 12].

VR could serve as an adjunctive therapy to SVR and may address adherence issues while improving outcomes. VR is defined as technology that immerses the wearer into an interactive environment that mimics reality [13]. This technology has been used in research as an adjunct to SVR sessions in both hospital and home settings. Studies of in hospital VR in patients with vestibular disease have involved the completion of SVR exercises while immersed in a VR environment with or without additional co-interventions [14–16]. In hospital VR has been shown to successfully improve stability, reduce dizziness, enhance quality of life, and reduce visual vertigo symptoms in these patients [14–16]. Recent meta-analyses support hospital VR as an effective and well tolerated intervention for vestibular disorders [17, 18]. Home based VR (HBVR) studies have required patients to play a 3D game utilizing a head mounted display (HMD) device in addition to completing in-clinic SVR and at home exercises [19, 20]. The addition of the HBVR gaming procedure to SVR significantly improved vestibular ocular reflex (VOR) gain, stability, balance confidence and patient quality of life compared to SVR and at home exercises [18–20].

HBVR video game exercises are thus a promising adjunct to SVR. However, there is a paucity of literature on which VR games are best suited for SVR, with Micarelli et al. utilizing a point-of-view VR racing game based on their judgment that it replicated SVR exercises [19, 20]. However, their video game selection was not conducted systematically and there is no consensus on what types of commercially available VR video games best replicate SVR exercises. Therefore, the purpose of this study was to determine which commercially available VR video game is most congruent with SVR exercises for peripheral vestibular disorders.

## Methods

### Video game selection

Virtual reality is defined as the immersion of the user in a digital environment that mimics the real world [13]. In contrast, augmented reality alters reality by projecting computer-generated sound, text and graphics onto the user’s natural visual and auditory fields [21]. Previous studies demonstrated that at home virtual reality racing games were effective as adjunctive vestibular rehabilitation [19, 20], hence racing games were the type of virtual reality games adopted for this study. Virtual reality video game selection was conducted systematically by searching “virtual reality racing” on the iOS App Store in March 2022. Video games were eligible if they were considered virtual reality point-of-view racing games that were free, had smooth functionality and were compatible with both iOS and Android. Video games were excluded if they required a joystick or were augmented reality video games. These video games were played on an iOS device placed in VR Shinecon G10 Virtual Reality Glasses. Copyright © (2022) Shinecon (Dongguan, China).

### Survey design

**Fig. 1:**
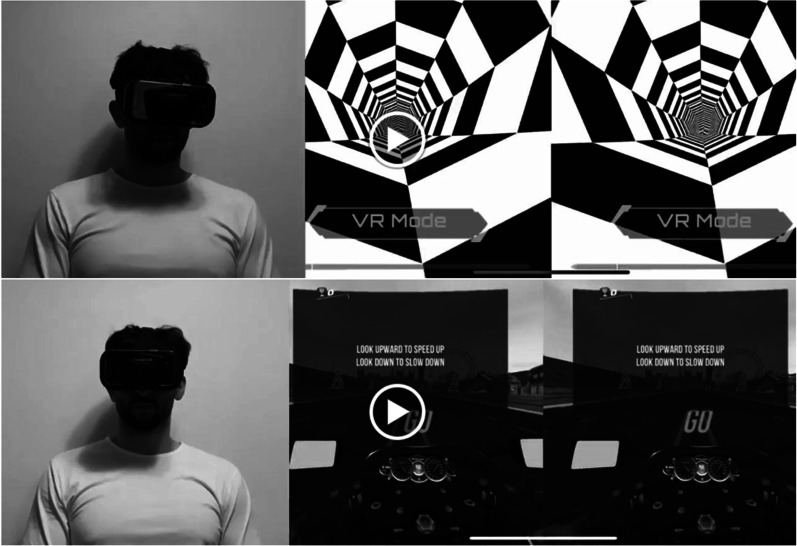
VR X-Racer and virtual reality tunnel racing and VR real world bike racing games. Anterior view of the user and the point-of-view of the user is displayed

**Fig. 2:**
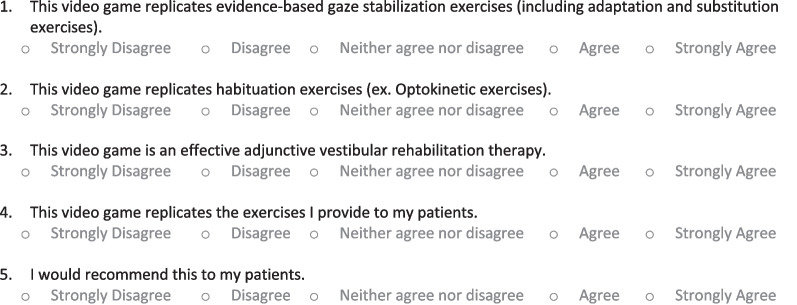
Virtual reality video game validation survey

Sample videos of a member of the research team playing the eligible video games along with a screen recording of the video game itself were included in the survey (Fig. 1) (https://www.youtube.com/watch?v=79q7bh3PbsA&ab_channel=AustinHeffernan). Physiotherapists were asked to watch each game and provide a response on a five-point Likert scale to the statements listed in Fig. 2. The five-point Likert-scale responses were scored as follows: strongly disagree (1 point), disagree (2 points), neither agree nor disagree (3 points), agree (4 points), and strongly agree (5 points). Surveys were delivered using the Qualtrics Software, Version 0822 of Qualtrics. Copyright © (2022) Qualtrics (Provo, UT).

### Physiotherapist recruitment

Physiotherapist recruitment was conducted using the Physiotherapy Association of British Columbia (PABC) website. Physiotherapists who self-identified as having training and/or experience in SVR were identified using the PABC website “Find a Physio” function. Eligible physiotherapists who held valid licensure with the College of Physiotherapists of British Columbia, were actively practicing, and who provided SVR were contacted through email to participate in the survey.

### Statistical analysis

Statistical analyses were performed using IBM SPSS Statistics version 24. Medians and interquartile ranges are reported for the scored physiotherapists’ responses to each video game’s survey statement and for global scores. Global scores were calculated for each survey, which is defined as the sum of the scored responses to each survey statement. A Friedman Two-Way ANOVA (*a-priori* alpha = 0.05) was used to compare the scored physiotherapists’ survey responses given for each of the video games assessed. If a statistically significant difference was identified, a paired samples sign test was used as a post hoc exploratory procedure to identify which video games differ based on the physiotherapists’ responses. Significance values for the sign test were adjusted by the Bonferroni correction for multiple tests, resulting in a significance level set at *p* < 0.002.

## Results

The search yielded 58 games, of which 4 games met the eligibility criteria, namely VR Tunnel Race (VRTR), VR Real World Bike Racing (VRWBR), Derby VR (DVR) and VR X-Racer (VRXR). A total of 237 physiotherapists were contacted and 40 (17%) consented to complete the survey in full. Survey results indicated that the largest fraction of physiotherapist responses was “agree” for VRXR, DVR, VRTR, and VRWBR replicating gaze stabilization exercises, matching habituation exercises, being effective adjunctive therapies and for recommending these games to their patients (Table 1). Additionally, the largest proportion of physiotherapists agreed that DVR, VRTR and VRWBR replicated physiotherapist prescribed exercises, however physiotherapists opinions on VRXR replicating physiotherapist prescribed exercises were almost evenly divided between disagreed (32.5%) and agreed (30%) (Table 1).

**Table 1:** Survey subscale response percentages for each of the four virtual reality video games

Statement	Game	Response				
		Strongly disagree	Disagree	Neither	Agree	Strongly agree
Replicates gaze stabilization exercises	VRXR	12.5	25	15	40	7.5
	DVR	7.5	20	10	50	12.5
	VRTR	7.5	30	12.5	37.5	12.5
	VRWBR	5	15	20	37.5	22.5
Replicates habituation exercises	VRXR	0	5	15	60	20
	DVR	0	5	25	57.5	12.5
	VRTR	0	0	10	45	45
	VRWBR	0	7.5	15	62.5	15
Effective adjunct therapy	VRXR	2.5	15	22.5	50	10
	DVR	2.5	10	32.5	47.5	7.5
	VRTR	2.5	2.5	20	60	15
	VRWBR	0	7.5	22.5	60	10
Replicates physiotherapist prescribed exercises	VRXR	12.5	32.5	25	30	0
	DVR	5	30	22.5	40	2.5
	VRTR	5	25	22.5	37.5	10
	VRWBR	2.5	25	17.5	45	10
Would you recommend it to your patients?	VRXR	10	12.5	30	37.5	10
	DVR	2.5	30	27.5	32.5	7.5
	VRTR	5	17.5	20	42.5	15
	VRWBR	0	15	22.5	52.5	10

*DVR* Derby Virtual Reality, *PT* Physiotherapist, *VR* Virtual Reality, *VRTR* Virtual Reality Tunnel Racing, *VRWBR* Virtual Reality Real World Bike Racing, *VRXR* Virtual Reality X-Racer

Among all four games, Friedman Two-Way ANOVA demonstrated significant differences in gaze stabilization exercise replication (*p* = 0.013), habituation exercise replication (*p* < 0.001), effective adjunctive therapy (*p* = 0.045) and physiotherapist prescribed exercise replication scores (*p* < 0.001) (Table 2). In terms of collated median global scores, VRTR and VRWBR were tied for the highest median score of 18 (Table 2). Paired samples sign test analysis indicated that VRTR replicated habituation exercises significantly (*p* < 0.001) better than DVR however VRTR and VRWBR did not differ significantly in their ability to replicate habituation exercises (Table 3). While VRWBR replicated physiotherapist-prescribed exercises significantly (*p* < 0.001) better than VRXR there was once again no discernable difference between VRWBR and VRTR (Table 3).

**Table 2:** Survey subscale median values and confidence intervals for all four virtual reality racing games

Subscale	VRXR [Median (IQR)]	DVR [Median (IQR)]	VRTR [Median (IQR)]	VRWBR [Median (IQR)]	Friedman’s ANOVA *p*-value
Replicates gaze stabilization exercises	3.00 (2.00)	4.00 (2.00)	3.50 (2.00)	4.00 (1.00)	0.013*
Replicates habituation exercises	4.00 (0.00)	4.00 (1.00)	4.00 (1.00)	4.00 (0.00)	< 0.001*
Effective adjunctive therapy	4.00 (1.00)	4.00 (1.00)	4.00 (0.25)	4.00 (1.00)	0.045*
Replicates exercises prescribed by PT	3.00 (2.00)	3.00 (2.00)	3.00 (2.00)	4.00 (2.00)	< 0.001*
Would recommend to patient	3.00 (1.00)	3.00 (2.00)	4.00 (1.00)	4.00 (1.00)	0.150

*DVR* Derby Virtual Reality, *IQR* Interquartile range, *PT* Physiotherapist, *VR* Virtual Reality, *VRTR* Virtual Reality Tunnel Racing, *VRWBR* Virtual, Reality Real World Bike Racing, *VRXR* Virtual Reality X-Racer

*A-priori alpha value = 0.05 for Freidman’s Two-Way ANOVA

**Table 3:** Post-hoc pairwise sign test results values for all four virtual reality racing games

Subscale	VRXR (A) versus DVR (B)	VRXR (A) versus VRTR (C)	VRXR (A) versus VRWBR (D)	DVR (B) versus VRTR (C)	DVR (B) versus VRWBR (D)	VRTR (C) versus VRWBR (D)
Replicates gaze stabilization exercises	A > B = 4	C < A = 5	A > D = 3	C < B = 10	D < B = 8	D < C = 4
	A < B = 12	C > A = 9	A < D = 17	C > B = 6	D > B = 10	D > C = 11
	Tie = 24	Tie = 26	Tie = 20	Tie = 24	Tie = 22	Tie = 25
	*p* = 0.077	*p* = 0.424	*p* = 0.003	*p* = 0.454	*p* = 0.815	*p* = 0.118
Replicates habituation exercises	A > B = 9	C < A = 3	A > D = 9	C < B = 1	D < B = 4	D < C = 16
	A < B = 2	C > A = 15	A < D = 6	C > B = 19	D > B = 7	D > C = 3
	Tie = 29	Tie = 22	Tie = 25	Tie = 20	Tie = 29	Tie = 21
	*p* = 0.065	*p* = 0.008	*p* = 0.607	*p* = < 0.001*	*p* = 0.549	*p* = 0.004
Effective adjunctive therapy	A > B = 10	C < A = 7	A > D = 6	C < B = 4	D < B = 5	D < C = 6
	A < B = 7	C > A = 16	A < D = 13	C > B = 13	D > B = 12	D > C = 4
	Tie = 23	Tie = 17	Tie = 21	Tie = 23	Tie = 23	Tie = 30
	*p* = 0.629	*p* = 0.093	*p* = 0.167	*p* = 0.049	*p* = 0.143	*p* = 0.754
Replicates exercises prescribed by PT	A > B = 5	C < A = 6	A > D = 3	C < B = 11	D < B = 7	D < C = 6
	A < B = 15	C > A = 19	A < D = 22	C > B = 14	D > B = 14	D > C = 9
	Tie = 20	Tie = 15	Tie = 15	Tie = 15	Tie = 19	Tie = 25
	*p* = 0.041	*p* = 0.015	*p* = < 0.001*	*p* = 0.690	*p* = 0.189	*p* = 0.607
Would recommend to patient	–	–	–	–	–	–

*DVR* Derby Virtual Reality, *PT* Physiotherapist, *VR* Virtual Reality, *VRTR* Virtual Reality Tunnel Racing, *VRWBR* Virtual Reality Real World Bike Racing, *VRXR* Virtual Reality X-Racer

*A-priori Bonferroni corrected two-tailed *p* value < 0.002

## Discussion

This is the first study that assessed licensed physiotherapists’ opinion on the vestibular rehabilitation potential of commercially available virtual reality videos games. They determined that VRWBR and VRTR were the two VR video games with the highest vestibular rehabilitation potential. Deciding between these two games could not be done based on study results and statistical analyses alone. While this study did not seek to determine user’s preferences for different videogames, their preferences will likely play a large role in treatment adherence, especially for older patients who are known to report reduced usability of new gaming technology [22]. One of the investigators (AH) who tried all videogames assessed, found VRWBR easier to use than VRTR on an iOS device, suggesting that VRWBR is the preferable game of choice for further clinical trial analysis in a cohort of older patients. Virtual reality has previously been shown to improve enjoyment and motivation during vestibular rehabilitation in young and middle-aged adults and thus has the potential to increase treatment adherence [18, 23].

For many chronic peripheral vestibular disorders, it is established that vestibular rehabilitation improves symptom scores and quality of life, however studies comparing the relative importance of gaze stabilization, habituation, and substitution exercises for specific vestibular pathologies are scarce and of poor quality [24, 25]. VRTR, in contrast to VRWBR, lacks the ability to alter the amount of optokinetic stimulation which prevents progressive grading of exercise difficulty, which is considered a therapeutic program requirement for effective recovery [26]. Additionally, this lack of grading could cause it to be visually over stimulating especially in patients who start this treatment soon after the onset of dizziness or vertigo symptoms. This may lead to reduced adherence to virtual reality vestibular therapy. These considerations together suggest that VRWBR is the preferred game of choice for clinical trialling.

Gaze stabilization exercises involve head movements in the vertical plane (pitch) and horizontal plane (yaw) to induce vestibular adaptation and to achieve substitution through enhanced VOR gain [12]. These exercises are used as the foundation of vestibular rehabilitation for bilateral and unilateral vestibular hypofunction [27–29]. In contrast to standard gaze stabilization exercises, both VRWBR and VRTR utilize user pitch head movements and movements in a sagittal plane (roll) to control the game. This difference could result in less effective vestibular substitution due to roll movements eliciting less enhancement in VOR gain [30]. Despite this, the combination of these movements and optokinetic stimulation from VRTR or VRWBR could benefit patients suffering from peripheral vestibular pathologies as the combination of habituation, adaptation and substitution exercises have been shown to elicit maximum benefit [25, 27]. This is evidenced by Micarelli et al. 2017 who demonstrated that a user’s pitch and roll movement based virtual reality video game combined with SVR improved dizziness handicap scores significantly more than SVR alone [19].

The demonstrable differences between some virtual reality programs, and SVR leaves the opportunity for innovation. Recently, a smartphone-based gaming system for vestibular rehabilitation has been developed which consists of two games with graded levels of difficulty that utilize optokinetic stimulation and discrete head movements in the pitch and yaw planes to achieve rehabilitation [31]. The games’ difficulty is determined by a performance-based algorithm [31]. This system was determined to be useable and safe to use in patients with unilateral vestibular dysfunction, however a randomized controlled trial testing its efficacy as an adjunct or sole treatment for chronic peripheral vestibular pathologies is lacking. This gaming system is a preliminary step in the development of an additional and more motivating treatment option for patients diagnosed with a chronic peripheral vestibular disorder. The results of the current study will determine how the assessed localization of vestibular pathology impacts the therapeutic effect of at-home adjunct virtual reality therapy. This will better inform the development and implementation of at-home virtual reality-based treatments for vestibular pathologies.

This study introduces the option of using VRWBR as an adjunctive treatment, however there are limitations to the conclusions drawn. The study by design is unable to provide a robust clinical recommendation for the use of VRWBR. A prospective randomized controlled clinical trial of VRWBR is required to arrive at a robust recommendation. The results are hindered by the lack of validated surveys that assess video games for their vestibular rehabilitation potential. The survey utilized in this study was created by medical students, a neurotologist, and a physiotherapist with a special interest in vestibular rehabilitation and was not subjected to validation testing prior to its use. The physiotherapists based their assessments on video recordings of an individual playing each video game; they did not personally use each video game. It is uncertain how this affects the accuracy of their responses. The licensed physiotherapists surveyed were recruited through the PABC website where physiotherapists can self-select interest/training areas. No attempt was made to select physiotherapists based on objective measures of vestibular education, knowledge, or documented experience. The low response rate is however likely a reflection of participant physiotherapists self selection as Balance and Dizziness Canada lists only 28 therapists in British Columbia who have completed at least one competency-certified formal exam or have taken multiple in-person post-professional courses without formal exams [32]. Future studies that survey objectively verified vestibular physiotherapists using a validated virtual reality video game questionnaire and that provides respondents with direct exposure to the games are needed to confirm our findings.

## Conclusion

In conclusion, according to the opinions of licensed physiotherapists, VRTR and VRWBR are the most congruent VR video games to SVR. These video games replicate both habituation and gaze stabilization exercises, could be effective adjunctive therapies to vestibular rehabilitation and would be recommended by physiotherapists to their patients. VRWBR is preferable to VRTR with respect to ease of use and the ability to alter the amount of optokinetic stimulation. Prospective studies are needed to confirm the efficacy of these videos games and to determine if they could be used as solitary treatments. These games could lay the foundation for the development of virtual reality video games that replicate vestibular rehabilitation exercises.

## Data Availability

The datasets used and/or analyzed during the current study are available from the corresponding author on reasonable request.

## Abbreviations

DVR: Derby VR
HMD: Head mounted display
PABC: Physiotherapy Association of British Columbia
SVR: Standard vestibular rehabilitation
VR: Virtual reality
VOR: Vestibular ocular reflex
VRTR: VR Tunnel Race
VRWBR: VR Real World Bike Racing
VRXR: VR X-Racer
